# Persister Cells – a Plausible Outcome of Neutral Coevolutionary Drift

**DOI:** 10.1038/s41598-018-32637-2

**Published:** 2018-09-25

**Authors:** T. M. Khlebodarova, V. A. Likhoshvai

**Affiliations:** Department of Systems Biology, Institute of Cytology and Genetics, Russian Academy of Sciences, Siberian Branch, Novosibirsk, Russia

## Abstract

The phenomenon of bacterial persistence – a non-inherited antibiotic tolerance in a minute fraction of the bacterial population, was observed more than 70 years ago. Nowadays, it is suggested that “persister cells” undergo an alternative scenario of the cell cycle; however, pathways involved in its emergence are still not identified. We present a mathematically grounded scenario of such possibility. We have determined that population drift in the space of multiple neutrally coupled mutations, which we called “neutrally coupled co-evolution” (NCCE), leads to increased dynamic complexity of bacterial populations via appearance of cells capable of carrying out a single cell cycle in two or more alternative ways and that universal properties of the coupled transcription-translation system underlie this phenotypic multiplicity. According to our hypothesis, modern persister cells have derived from such cells and regulatory mechanisms that govern the consolidation of this phenomenon represented the trigger. We assume that the described type of neutrally coupled co-evolution could play an important role in the origin of extremophiles, both in bacteria and archaea.

## Introduction

The phenomenon of bacterial persistence (persister bistability) – a non-inherited antibiotic tolerance in a minute fraction of the cellular population^1–11^ is a proven example of the existence of phenotypic multiplicity in a genetically homogeneous population of bacterial cells^12^. This phenomenon was discovered in 1944^13^ and its essence lies in the fact that an antibiotic-sensitive population of bacterial cells practically always, even after prolonged antibiotic therapy, contains a very small number of cells (10^−3^–10^−6^) possessing tolerance to the antibiotic and therefore is capable of restoring cellular population after withdrawal of treatment. However, such cells do not convey this property of tolerance to daughter cells. The restored population of bacterial cells is also antibiotic-sensitive, like the original population.

Until this century, there were practically no systematic theoretical and practical studies dedicated to this phenomenon. It might be possible that lack of interest in this phenomenon was due to both low-frequency occurrence and problematic identification of these cells as well as the fact that replica method that allowed demonstrating preexistence of mutant antibiotic-resistant cells in bacterial populations^14^ was developed shortly after the discovery of persister cells. As a result, up to the end of the 20^th^ century, main efforts have been directed towards addressing mutational mechanisms of the antibiotic resistance formation. At present, resistant and persister cells are differentiated by the ability of their daughter cells to grow in the presence of antibiotics: daughter cells of resistant cells do grow whereas those of persister cells do not^7^.

Systematic studies on persister cells have began only in the 2000s after the development of approaches allowing their identification and isolation^12,15,16^, although first mutation that significantly increases the frequency of persister formation was described as early as the 1980s^17^. Currently, persister cells are considered one of the reasons for the chronic course of many infectious diseases^2–4,6^, ets.

Studies on persister cells have demonstrated that they constantly appear in the exponentially growing cell culture; differ functionally from these cells and from the stationary-phase cells; and are characterized by the low level of protein synthesis, small size, and slow growth^12,15,16,18^. It was found that the number of persister cells increases during transition from exponential to stationary phase of growth and under oxidative stress; under the action of DNA-damaging agents; during growth in nutrient-poor medium; and during transition from freely-suspended cells to a film culture^3,19–21^. These data suggest that bacterial persistence can be a reflection of a more general strategy of cellular adaptations to external influences, rather than only in relation to antibiotics.

Analysis of the functional activity of persister cells revealed a significantly higher expression of genes belonging to the stress response systems, including the toxin-antitoxin (TA) systems^8,11,15,16^. The study of mutant genes belonging to various TA systems confirmed their participation in the formation of persister cells^12,22–25^. Although it is obvious that formation of some portion of persisters occurs due to functioning of TA systems, it is necessary to take into account that mutations in individual genes and gene regulators of TA systems reduce the frequency of persisters, but do not eliminate them from the population^12,22–24^. In other species of bacteria deletions in TA modules do not affect the frequency of persisters^25^. Moreover, mutations in metabolic genes affect the frequency of persister cells in bacterial populations^26,27^. If we take into account that nonspecific suppression of transcription and translation has the greatest effect on the frequency of persister cells^28^, it should be recognized that a certain proportion of persister cells might arise due to some universal processes common to all bacterial species that allow cells with different levels of metabolism, high or low, to be formed in genetically homogeneous populations.

To test this assumption, we have previously developed a simple model of the cell cycle with a synchronous initiation of replication and uniform division, which also lacked any regulatory contours that create internal conditions for bistability^29^. The main result obtained was that in the absence of any regulatory influences two different stable scenarios of the cell cycle were detected in the model. The first scenario we designated as R-phenotype. It was characterized by a short breeding cycle, large volume of a newborn ≪cells≫ (with quotation marks we emphasize that we talk about modeled, not natural cells), intensive replication, and active metabolism. ≪Cells≫ developing via second scenario had relatively small size and were characterized by slow growth and low levels of metabolism and replication. They were designated as cells bearing the S-phenotype. Characteristics of the R-phenotype were similar to those of rapidly growing cells of *E*. *coli*, *Bacillus subtilis*, and *Salmonella typhimurium* developing in a rich medium^30^. The S-phenotype was surprisingly similar in its characteristics to the so-called persistent phenotype of natural cells^12,18^.

Results obtained earlier^29^ raised an important question: is it possible that cells capable of carrying out a single cell cycle in two alternative ways-phenotypes are the ancestors of modern persister cells capable of switching from active development to the state of a persister cells under stress conditions? However, this question has not been addressed in^29^.

In this paper we give an answer to this question. Based on the analysis of the minimal model of a single cell cycle of a self-reproducing system we drew a conclusion that nonlinear properties of the coupled transcription-translation system represent a molecular genetic basis for the phenotypic multiplicity of the cell cycle. We theoretically substantiated that biochemical nature of the living organisms implies the possibility of neutral evolution of their systems in the space of multiple coupled mutations, which we designated as neutral coupled co-evolution. We identified sufficient conditions under which, at the stage of maximum adaptability, the process of neutral coupled co-evolution naturally leads to the appearance of cells capable of carrying out a single cell cycle in several different ways (phenotypes). We concluded that phenotypic multiplicity can be inherent in almost any unicellular organisms and could had occurred multiple times as early as during the archetypal stages of the evolution of unicellular organisms. Perhaps even earlier than the basic principles of hereditary information storage and transfer (the coupled transcription-translation system) had been formed, since the phenomenon of phenotypic multiplicity is a manifestation of autocatalytic properties of biochemical systems.

We believe that the phenomenon of persister bistability in modern cells is the result of phenotypic multiplicity consolidation at the genetic level that took place in ancient cells in the process of population drift in the space of multiple neutrally coupled mutations, which we called “neutrally coupled co-evolution” (NCCE).

## Results

### Phenotypic multiplicity of the cell cycle as an outcome of the universal properties of the coupled transcription-translation system

Among all the subsystems existing in modern cells a special place belongs to the coupled transcription-translation system, since it accomplishes synthesis of protein molecules, which participate in absolutely all processes in a cell at all stages of the cell cycle.

Previously, we proposed the hypothesis of the origin of bimodal cellular distribution according to the growth rate, cell size, and metabolic level, which allows illustrating the characteristics of persister cells^12,18^ based on the basic properties of the transcription-translation system^29^, however mechanisms that underlie phenotypic multiplicity have not been studied.

For the purposes of our analysis, we note two key functioning features of the transcription and translation system. The first feature is that there is a certain group of proteins in a cell (denoted by *B*), which is part of the transcription and translation system and therefore these proteins participate directly in their own synthesis and synthesis of all the other proteins. This group of proteins is quite extensive: these are ribosomal proteins, RNA polymerases, aminoacyl-tRNA synthetases, initiation and termination factors of transcription and translation, and a number of others. For example, there are about 200 genes encoding proteins of this group in *E*. *coli*^31,32^. In fact, these proteins represent a class of self-replicating proteins and the dynamics of their autocatalytic synthesis determines the rates of all other processes, including DNA replication, cell metabolism, etc.

The second feature is that a certain group of proteins performs control over the cell cycle, which is normally accompanied by cell volume increase (denoted by H). Therefore, the cell volume growth rate depends on the concentration of such self-reproducing proteins as ribosomes, RNA polymerase, etc.

Protein degradation is an important element involved in the cell cycle regulation of modern cells, considering the external and internal environmental factors. But, for simplicity, we excluded protein degradation from consideration, although degradation reduces protein concentration and under certain conditions can lead to complex chaotic changes in intracellular protein concentrations^33^.

We assume that if we identify the mechanism for the formation of phenotypic multiplicity of the cell cycle, which does not include degradation processes, this would mean that protein degradation is not the essential phenomenon-forming factor, although it can play a similar role under certain conditions.

When investigating the conditions that generate phenotypic multiplicity of the cell cycle (for details, see Supplementary SI1), we found that if a protein does not belong to the group *B* its synthesis and dilution can act as factors that engender phenotypic multiplicity of the cell cycle only if there are specific feedback regulatory loops involved in its synthesis^34,35^. Otherwise, synthesis and dilution of proteins not belonging to the group *B* are not capable of generating phenotypic multiplicity, whereas synthesis and dilution of proteins from group *B* (RNA polymerases, ribosomal proteins) can potentially act as factors that engender phenotypic multiplicity of the cell cycle.

#### What are these mechanisms?

**Figure 1:**
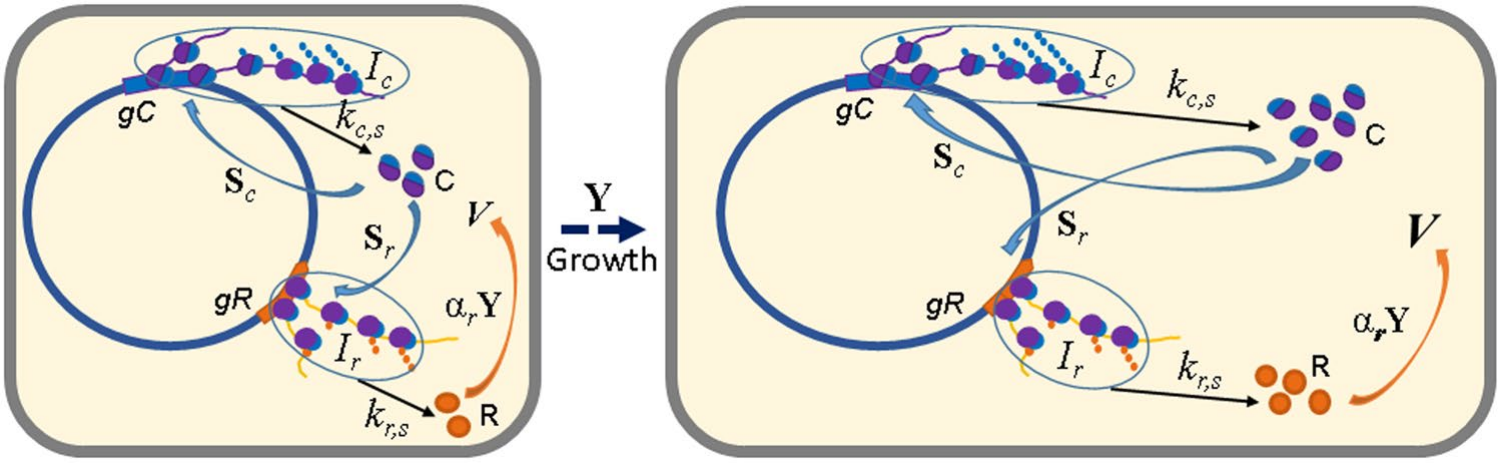
The diagram depicting processes considered in the simple model of the cell cycle. *V* – ≪cell≫ volume, С – generalized synthesis factor, R – generalized growth factor, **S**_c_ – synthesis initiation of the factor C, **S**_r_ – synthesis initiation of the factor R, *I*_c_ – polysomes that synthesize the factor C, *I*_r_ – polysomes that synthesize the factor R, *k*_*c*,*s*_ – rate constant for the synthesis of the factor C, *k*_*r,s*_ – rate constant for the synthesis of the factor R, **Y**- general law of ≪cell≫ growth, α_*r*_ – the number of growth factor molecules consumed during a single cell cycle. The growth process is schematically shown by the ≪cell≫ size increase from left to right.

To answer this question we considered the minimal model of a single life cycle of the simplest self-replicating ≪cell≫ system. Only two main processes were considered in the model: ≪cell≫ volume *V* growth (with quotation marks we emphasize that we talk about modeled, not natural cell) and the synthesis of two generalized factors, namely the synthesis factor C (analogue of the ribosome) and the growth factor R (analogue of the mass protein of the *E*. *coli* cell membrane – Lpp^36^). Factor C ensures synthesis of factor R, which is consumed during cell growth, as well as the autocatalytic synthesis of factor C molecules. The process of genome replication was not explicitly considered in the model, nor were considered the separate processes of transcription and translation and the formation of multimers of the active factors. In addition, processes of utilization/dissipation and metabolism, as well as the molecular genetic regulatory control circuits in single-cycle processes were not considered in the model. The diagram depicting processes considered in the simple model of the cell cycle is represented in Fig. 1.

The minimal model of a single cell cycle of a self-reproducing system is represented by the following system of ordinary differential equations:

1 $$\{\begin{array}{c}\frac{dV}{dt}={\bf{Y}}V,\\ \frac{dc}{dt}={k}_{c,s}{I}_{c}+(\,-\,{{\bf{S}}}_{c}+{k}_{c,s}{I}_{c})+(\,-\,{{\bf{S}}}_{r}+{k}_{r,s}{I}_{r})-\frac{dV}{dt}\frac{1}{V}c,\\ \frac{dr}{dt}={k}_{r,s}{I}_{r}-{\alpha }_{r}{\bf{Y}}-\frac{dV}{dt}\frac{1}{V}r,\\ \frac{d{I}_{c}}{dt}=-\,(\,-\,{{\bf{S}}}_{c}+{k}_{c,s}{I}_{c})-\frac{dV}{dt}\frac{1}{V}{I}_{c},\\ \frac{d{I}_{r}}{dt}=-\,(\,-\,{{\bf{S}}}_{r}+{k}_{r,s}{I}_{r})-\frac{dV}{dt}\frac{1}{V}{I}_{r}.\end{array}$$

where, *k*_*c*,*s*_ and *k*_*r*,*s*_ – rate constants for factor C and factor R synthesis, respectively; *V* – ≪cell≫ volume; *c* – total concentration of free molecules of the synthesis factor С and molecules involved in the initiation of factor C synthesis; *r* – concentration of free molecules of the growth factor R; α_*r*_ – the number of growth factor molecules consumed during a single cell cycle; **Y** – general law of cell growth; **S**_*c*_ and **S**_*r*_ – synthesis initiation rates for synthesis and growth factors, respectively; *I*_*c*_, *I*_*r*_ – pools of synthesis factors involved in the elongation of synthesis and growth factors, respectively. Here and further in the text, the laws of growth and synthesis initiation are indicated in bold non-italic font, names of factors – in non-italic font, concentrations, constants, and indices - in italic font.

**Figure 2:**
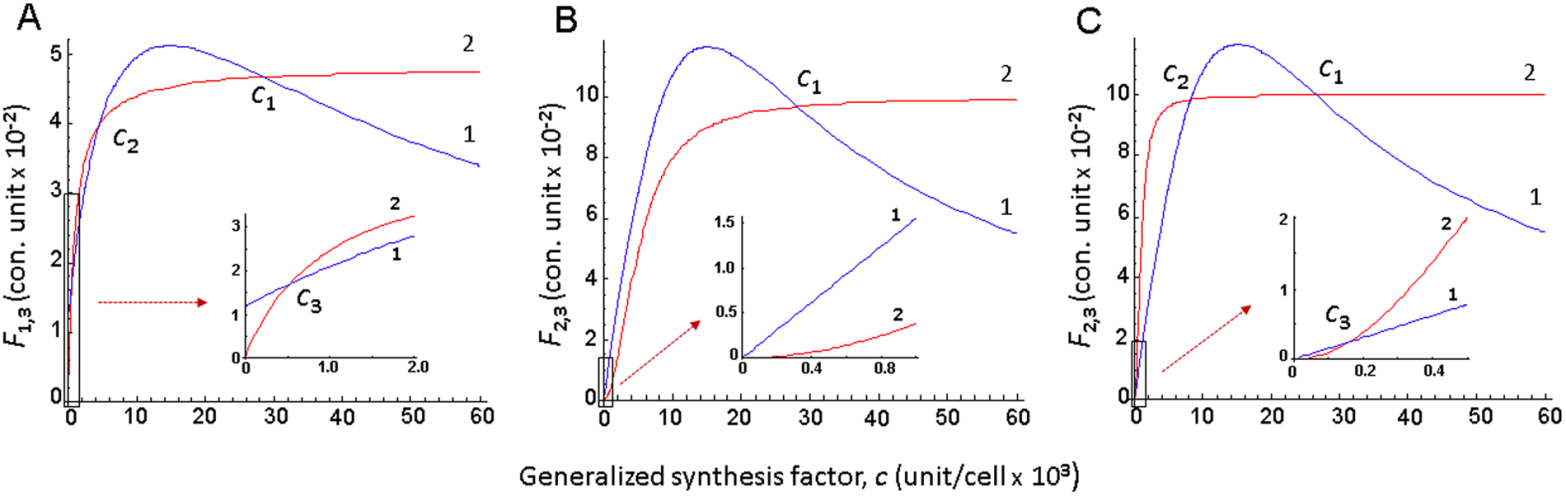
Correlation between relative rates of factor *c* synthesis and dilution and its concentration in the ≪cell≫. (**A**) Equations (8 and 11), curve 1 – relative rate of synthesis **s**_*c*_/C (*F*_1_); curve 2 – relative rate of dilution **s**_*r*_/α_*r*_ (*F*_3_); calculations were made for *k*_*e*_*/n* = 1.35 min^−1^, α_*r*_ = 500000 unit/cell, *K*_*r*_ = 1000 unit/cell. (**B**,**C**) Equation (20), curve 1 – relative rate of synthesis **S**_*c*_/*c* (*F*_2_), curve 2 – relative rate of dilution **S**_*r*_/α_*r*_ (*F*_3_), calculations were made for α_*r*_ = 250000 unit/cell, *K*_*r*_ = 5000 unit/cell (**B**) *K*_*r*_ = 1000 unit/cell (**C**). Values of the remaining parameters are *k*_*c*_ = 3500 unit/cell/min, *k*_*r*_ = 25000 unit/cell/min, *K*_*c*_ = 15000 unit/cell. Abscissa – concentration of the generalized synthesis factor *c* (unit/cell), ordinate – values of the relative rates of synthesis and dilution (con. unit).

Next, we introduced a number of simplifications and by standard calculations the problem of phenotypic multiplicity in the model (1) was reduced to the analysis of positive solutions to equation (8) (for details see section “Methods”). The problem of the number of positive solutions to equation (8) is also predisposed by the type of functions **S**_*c*_, **S**_*r*_ that describe nonlinear formation of active complexes between generalized synthesis factors and their binding sites located on the corresponding mRNA molecules (analogs, SD sites). Analysis of the possible types of functions **S**_*c*_, **S**_*r*_ (see section “Methods”) revealed that if elongation stage is considered, phenotypic multiplicity of a single cycle is realized even with the simplest mechanism of factors synthesis initiation (equation (9)), in which the active form of the synthesis factor *s* is identical with the monomer *c*. In this case, synthesis functions have the form of monotonically increasing functions (equation (11)). Corresponding calculations demonstrating the multiplicity of solutions to equation (8) with functions of the form equation (11) are given in Fig. 2A.

If the elongation stage is excluded from the model, the plausibility of phenotypic multiplicity arises when quadratic nonlinearities are introduced into the mechanism of factor’s synthesis. Similar properties are possessed by the simplest synthesis initiation scheme (equation (9)), in which the active form of the synthesis factor *s* is a dimer *с*^2^ and not a monomer, which is not a limitation for the translation initiation system. In this case, instead of equations (8 and 11) we get the balance equation and synthesis function equation (20).

It can be seen from Fig. 2 that equation (20) has one positive root for certain parameter values (Fig. 2B) and three positive roots – for other (Fig. 2C).

Thus, the obtained results demonstrate that the ability of self-replicating systems to carry out a single cell cycle in at least two different ways is a result of the synthesis system that, during a single cycle, autocatalytically synthesizes elements (factors) of itself and elements (factors) of the growth subsystem. Moreover, the molecular prerequisites for the formation of the ability of the ≪cell≫ to carry out a single cycle in several alternative ways already arise if biochemical mechanism of the protein synthesis has a minimum quadratic level of nonlinearity that is multiply exceeded in modern cells.

In addition, since the transcription-translation apparatus is present in all modern cells without exception and is identical in the considered details, all cells, regardless of their belonging to the kingdom of bacteria, archaea, or eukaryotes, have a potential for the bistability formation. This illustrates that formation of the cell cycle phenotypic multiplicity is universal.

### The role of neutral coupled co-evolution in the appearance of cells possessing phenotypic multiplicity of a single cell cycle

Taking into account the above theoretical results demonstrating that the coupled transcription-translation apparatus being a self-reproducing system that was formed at the earliest stages of cellular evolution represents an apparatus that naturally carries properties ensuring realization of phenotypic multiplicity of a single cell cycle in living organisms, it is fair to assume that molecular prerequisites for the formation of bistability are of very ancient origin.

In this connection, the question arises as to what patterns of evolution of living systems could immanently bear conditions for the emergence of cell cycle bistability.

To address this question, in this section we studied the outcome of the evolutionary process that facilitates the selection of cells with increased adaptation to living conditions. We proceeded from the firmly established fact that mutations due to errors during replication represent a constant source of variability and material for evolutionary selection. Within the framework of the present work, we considered only mutations that change the parameter values of the rates of molecular processes, but do not have an impact on their mechanisms. Taking into account that mutations are rare, we compared cells with cell cycle in an equilibrium state. Let us assume that external conditions are identical and constant for all cells and their descendants and that probability of spontaneous death is the same for all cells in the entire range of existence. Under such assumptions, the specific cell growth rate W that is reciprocal of the cell cycle duration represents a natural measure of adaptation. And evolution of an individual cell towards improving the adaptability is regarded as the succession of descendants in the direction of decreasing the duration of the cell cycle T, or increasing its reciprocal value W = 1/T.

Analysis of functional properties of the adaptability functional W was carried out in the model (13) of a single life cycle of a self-reproducing system in which the elongation stage was not considered. It was demonstrated that the adaptability functional W has a single global maximum, which under certain conditions is realized not at a single point, but in some nontrivial parametric area, which can be designated as the area of neutrality. The analysis has revealed that this area has subareas with multiple phenotypes of a single cell cycle, which are realized under the relative lack of nutritional resources. Moreover, parameters of the modern actively growing *E*. *coli* cell^36–40^ completely satisfy the conditions for the realization of phenotypic multiplicity (for details see Supplementary SI2).

#### Neutral drift in the parametric space of coupled mutations

Let us consider the evolution of cells in the light of the data presented above.

What happens to the cell that has reached maximum adaptability? Evidently, evolution that previously maintained a directed selection of mutations that improve cell adaptability is no longer possible for such cells. That is, for a cell in a state of maximum adaptability the driving type of evolution changes to the stabilizing type. This means that vast majority of mutations would now decrease the adaptability of cells and their descendants carrying such mutations would be eliminated from the population. As a result, neutral mutations would be the only source of genetic variability in such cells. M.Kimura and colleagues have studied the random genetic drift acting on selectively neutral mutants and named this process “neutral evolution”^41,42^. In their sense, single neutral mutations do not lead to the appearance of new properties, but possess this potential when coupled with non-neutral mutations.

Along with single mutations, multiple (double, triple, etc.) mutations occasionally appear, which are definitely harmful one at a time, but their combined effect might be neutral. Cells that carry such combinations of mutations would remain maximally adapted and would not be subject to directed evolutionary selection. As a result, over the course of time, cell population with random genetic drift acting on both neutral mutations and multiple neutrally coupled mutations would go over all variants of multiple neutrally coupled mutations with a single probability.

An important question arises: are there neutrally coupled mutations in natural cells?

We firmly believe that there is no evidence for a negative answer to this question. Practically non-alternative positive answer follows from the obvious fact that cells represent biochemical reactors, in which a large number of reversible reactions occur simultaneously. And reversible reactions carry a natural mechanism for the emergence of neutrally coupled mutations. In order to justify this, it is sufficient to note that mutations that change the values of direct and inverse variations of ≪fast≫ reversible reactions in such a way that their ratio does not change are almost neutrally coupled.

The results presented in Supplementary SI2 and SI3 demonstrate that paired mutations, which change the parameter values of the rate constants for synthesis and dissociation of synthesis factors (*k*_*c*_, *K*_*c*_) in a certain coordinated way, possess properties of neutrality.

The condition for consistency was obtained from the analysis of the conditions, under which a given pair of parameters enter the adaptability functional W. Indeed, the pair (*k*_*c*_, *K*_*c*_) belongs to W not directly, but as part of the function S_*с*_, so if a maximally adapted ≪cell≫ has some fixed set of values (*k*_*c*,0_, *K*_*c*,0_), then any ≪cell≫ possessing different set of physiologically relevant values of the parameters *k*_*c*_ and *K*_*c*_ that satisfy the equality

2 $$\frac{{k}_{c}}{{K}_{c}^{2}+{c}^{2}}=\frac{{k}_{c,0}}{{K}_{c,0}^{2}+{c}^{2}}.$$

also represents a maximally adapted ≪cell≫.

Thus, this equation defines a parametric variety of consistent parameter values within which a neutral drift of ≪cells≫ occurs. That is, after the ≪cell≫ reaches the state of maximum adaptability, evolution of its descendants accumulating multiple mutations that do not violate the equality equation (2) would proceed in the neutrality regime. Moreover, if the nutritional resource (Δ_*z*_), which, as shown in Supplementary SI3, is a natural factor for the formation of phenotypic multiplicity of the cell cycle, is in a relative deficit, in the sense of satisfying the inequality Δ_*z*,*min*_ < Δ_*z*_ < Δ_*z*,*max*_, then the region of neutral coupled co-evolution for parameters (*k*_*c*_, *K*_*c*_) intersects with the region of phenotypic bistability. Under these conditions, descendants of the maximally adapted ≪cell≫ include cells possessing combinations of mutations ensuring the ability to implement a single cell cycle in at least two alternative ways.

## Discussion

What can we state about the origin of persister cells in terms of the theory on phenotypic multiplicity given above?

From the presented analysis, we concluded that universal nonlinear properties of the coupled transcription-translation system are the molecular basis for the emergence of the very possibility of carrying out the cell cycle not in one, but in several alternative ways (phenotypic multiplicity). Moreover, the minimality of the considered model is not critical for the conclusion drawn, since we previously observed a similar phenotypic multiplicity phenomenon for a more complex model of the cell cycle, which considered processes of replication, mRNA and protein synthesis, and a number of others^29^. Accordingly, we assume that phenotypic multiplicity is potentially inherent in all types of natural cells, both ancient and modern. We suggest that this property had appeared in the extreme antiquity, perhaps even earlier than the mechanisms of coupled transcription-translation system was formed, since the bistability property underlying the phenomenon of phenotypic multiplicity is inherent in simple autocatalytic systems.

From the minimal model analysis, we also deduced that nutritional deficiency is sufficient for realization of phenotypic multiplicity. Such conditions seem quite typical for natural habitats.

We have also investigated properties of the evolutionary process that facilitates the selection of cells best adapted to the environment. We used a reciprocal of the cell cycle duration as a measure of adaptability. We have found that adaptability in the space of values of evolving parameters has a single global maximum. However, under certain conditions (for example, relative nutritional deficiency), the global maximum is realized not for a single parametric point, but for some non-trivial variety of neutrally coupled mutations. As a result, further evolution of cells that have reached maximum adaptability proceeds in a neutral drift regime in this manifold.

This type of evolution, in fact, refers to the phenomenon of neutral evolution discovered and studied by M. Kimura and several other researchers^42–44^, the importance of which in the evolutionary process is greatly appreciated until now^45^. However, in its basis, this type of evolution is fundamentally different from the classical neutral evolution of Kimura^41,42^. The main difference is that this evolution takes place in the space of not single, but multiple mutations coupled according to the principle of neutral consistency. Each individual mutation in such a group (the minimum number of mutations is two) can be deleterious, but their combined effect on biochemical processes (in our case, the process of coupled transcription-translation) at the physiological level compensates for each other’s deleterious action and in relation to evolutionary selection at the level of adaptation they are neutral.

The possibility of compensating for deleterious mutations is observed in the catalytic properties of reversible biochemical reactions that underlie all cellular processes, and was previously demonstrated by M.V. Volkenshtein^46,47^ on the example of the simplest enzymatic reaction described by the Michaelis-Menten equation^48,49^. In a more general form, possibility of existence of compensatory mechanisms for minimizing the phenotypic manifestation of deleterious mutations was expressed by I.I. Shmalhausen back in 1946 (cited in^50^). Currently this type of compensation – via consistent multiple coupled mutations – has been demonstrated on the example of evolution of start codons and Shine-Dalgarno translation initiation sites^51^.

The new quality that we observed for this type of evolution, which we named ≪neutral coupled co-evolution≫, was the fact that in a population of ≪cells≫ carrying out a cell cycle in only one way (≪one genotype → one phenotype≫ development strategy), under certain conditions, the neutral drift ensures a virtually non-alternative appearance of an offspring with a capability of carrying out a cell cycle in several alternative ways (≪one genotype → several phenotypes≫ development strategy). That is, the type of ≪neutral coupled co-evolution≫ described in this paper has the potential of complicating the dynamic functioning of a population of living organisms. It is easy to assume that, in the course of evolution, more complex dynamic behaviour of living organisms led to the complication of their genetic programs as well as their morphological structures.

Taking together the results from both our studies^29^, we suggest that modern cells, which under stress conditions are capable of switching from active growth to a state with extremely low metabolic rates (persister cells)^12,18^ have originated from cells, which at a certain stage of evolution acquired the ability to implement a cell cycle in several alternative ways. In our opinion, the property of phenotypic multiplicity was useful for the survival of cell populations that periodically fell into strict conditions difficult for the survival of actively growing cells (R-phenotype according to^29^), but not critical for cells with low levels of metabolism (S- phenotype according to^28^). Later, the property of bistability, being evolutionarily advantageous, was consolidated via formation of special molecular genetic mechanisms, including toxin-antitoxin (TA) molecular triggers, which, under stress conditions, transfer cells from a stress-sensitive to a stress-resistant phenotype – a phenomenon, which we observe in modern bacterial species^12,22–25^.

Ways of molecular-genetic consolidation of phenotypic multiplicity, including via TA molecular triggers, are beyond the scope of this study and require further investigation. Nevertheless, we shall note that formation of new functionalities can be caused by the coupled transcription-translation system, whose components (RNA polymerases, ribosomes) are complex multimeric complexes that have the ability to interact with certain DNA or RNA sites. Because biochemical reactions are reversible, there is always a certain amount of free molecules of RNA polymerase subunits and ribosomes in the cell. If some of these molecules are inactive in a free state, but retain a certain affinity for their target sites, then they can act as repressors of this process, which means that TA systems could appear through the duplication of such genes: first duplication generates a global repressor of transcription (translation), and second – its own repressor.

Bearing in mind that variability of evolutionary paths is extremely prominent, the proposed theory does not contradict the possibility that ≪persister cells≫ are not direct descendants of ≪bistable≫ cells, but have another origin^52–54^. Including their origination via direct formation in initially ≪monophenotypic≫ cell population of trigger regulatory mechanisms controlling specific molecular genetic systems that promote formation and maintenance of bistability^55–59^.

Nevertheless, we note that the proposed theory explains such ≪hot≫ questions about the origin of persister cells as their ≪ineradicable≫ nature and constant presence in the population, as well as their non-inherited tolerance to stressful conditions during population recovery after stress elimination. The proposed theory is based on the phenomenon of neutral coupled co-evolution and explains the origin of cells possessing persistent phenotype on the basis of universal mechanisms of functioning of living systems. In this sense the proposed theory is universal and applicable to evolutionally distant prokaryotic species^12,15,16,18,60^.

**Figure 3:**
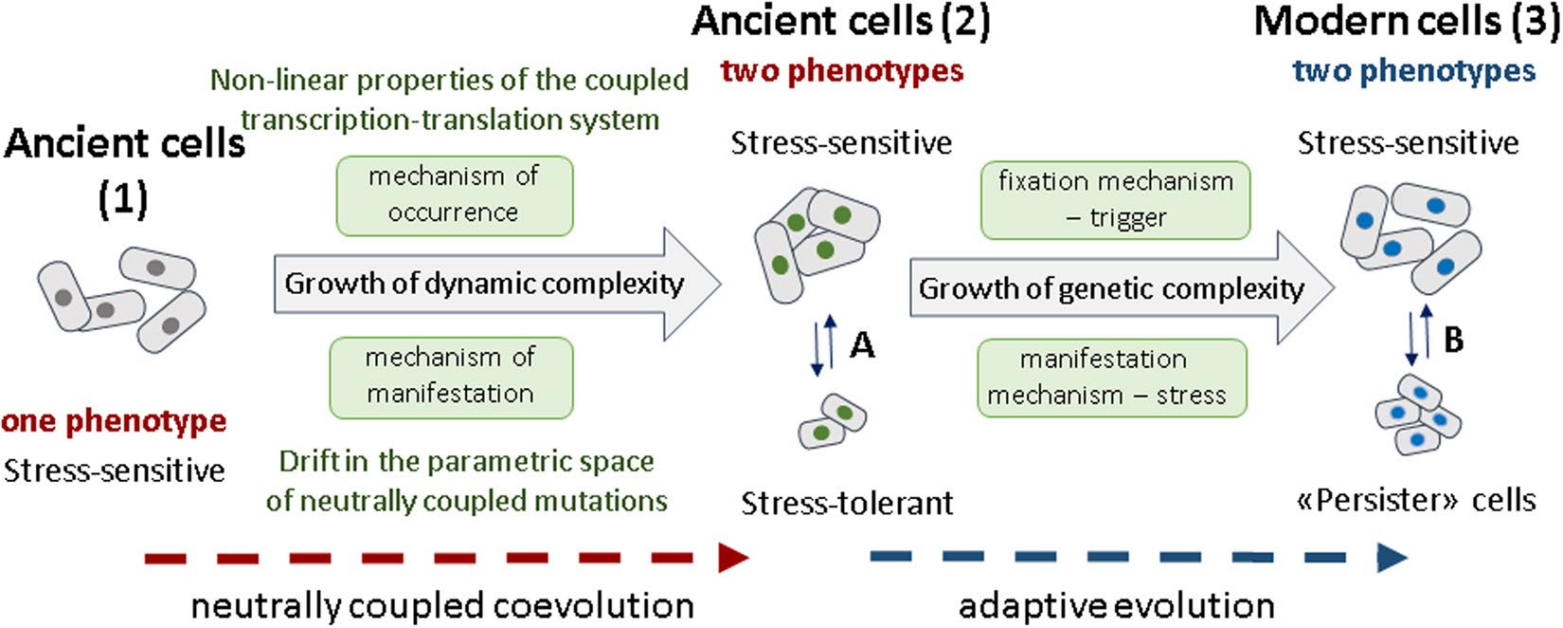
Scenario of the appearance of ≪persister≫ cells. As a result of the neutrally coupled co-evolution and due to nonlinear properties of the coupled transcription-translation system, a population of ≪ancient cells≫ (1) consisting of cells capable of carrying out a single cell cycle in one and only way gave rise to another population of ≪ancient cells≫ (2) consisting of cells of a qualitatively new type, namely capable of carrying out a single cell cycle in several alternative ways (phenotypes). The transition between phenotypes is a random event that occurs due to fluctuations in concentrations of the intracellular components during cell division. The population of ≪modern cells≫ capable of surviving under stress conditions (3) originated from the ≪ancient population≫ of cells (2), in which, in the course of adaptive evolution, regulatory contours of a trigger-type were formed and consolidated at the genetic level, which included both phenotypes in the norm of reaction. Drug-sensitive cells and ≪persister≫ cells of this population represent genetically identical cells that carry out a cell cycle in alternative ways^12^. The transition occurs under stress.

The results of our research are summarized in Fig. 3, which illustrates the scenario of development of phenotypic complexity in a cell population due to complications in the population functional dynamics and its drift in the space of multiple neutrally coupled mutations.

We assume that the ability to carry out a single cell cycle in several alternative ways could had been realized multiple times in the course of cellular evolution under changing external conditions. Existence of persister cells both in the kingdom of bacteria, and in the kingdom of achaea represents an indirect evidence in favor of this assumption^12,60^. Moreover, we do not exclude the possibility of independent evolution of alternative metabolic phenotypes under changing environments due to different resistance strategies in extreme conditions. That is how extremophiles and mesophiles could had driven apart during evolution, both in the kingdoms of archea and bacteria. The emergence of bacteria that form spores in response to unfavourable external conditions, in which metabolic activity is completely depleted, could also be easily explained by the assumption that cells consolidate the property of bistability via formation of specific molecular genetic mechanisms.

## Methods

All results of the present work were obtained by analytical methods. Specific logical and mathematical calculations are presented in this section, as well as in the ≪Results≫ section and in the Supplementary.

### Mathematical analysis of mechanisms providing a molecular genetic basis for the emergence of the possibility of the bistability phenomenon realization

In order to carry out such analysis, we introduced a number of simplifications and reduced the problem of phenotypic multiplicity in the model (1) to the analysis of positive solutions of cubic equation. We assumed that growth factor R is rapidly consumed during the cell growth while we ignore its dilution due to cell volume growth. We also assumed that the rate of cell growth is limited by the synthesis rate of the growth factor.

3 $${\bf{Y}}\approx \frac{{k}_{r,s}}{{\alpha }_{r}}{I}_{r}.$$

As a result, from system (2) we move to system (4)

4 $$\{\begin{array}{c}\frac{dV}{dt}=\frac{{k}_{r,s}}{{\alpha }_{r}}{I}_{r}V,\\ \frac{dC}{dt}={k}_{c,s}{I}_{c}-\frac{{k}_{r,s}}{{\alpha }_{r}}{I}_{r}C,\,C=c+{I}_{c}+{I}_{r},\\ \frac{d{I}_{c}}{dt}=({{\bf{S}}}_{c}-{k}_{c,s}{I}_{c})-\frac{{k}_{r,s}}{{\alpha }_{r}}{I}_{r}{I}_{c},\\ \frac{d{I}_{r}}{dt}=({{\bf{S}}}_{r}-{k}_{r,s}{I}_{r})-\frac{{k}_{r,s}}{{\alpha }_{r}}{({I}_{r})}^{2}.\end{array}$$

where *C* – total concentration of the synthesis factor C. Consider the last two equations of the equality:

5 $${I}_{c}=\frac{{{\bf{S}}}_{c}}{{k}_{c,s}+\frac{{k}_{r,s}}{{\alpha }_{r}}{I}_{r}},{I}_{r}=\frac{{{\bf{S}}}_{r}}{{k}_{r,s}+\frac{{k}_{r,s}}{{\alpha }_{r}}{I}_{r}}.$$

Express *I*_*r*_ through **S**_*r*_:

6 $${I}_{r}=\frac{{{\bf{S}}}_{r}}{\sqrt{\frac{{k}_{r,s}}{{\alpha }_{r}}{{\bf{S}}}_{r}+{(\frac{{k}_{r,s}}{2})}^{2}}+\frac{{k}_{r,s}}{2}}$$

Values of the synthesis rate constants for factors are assumed equal for all genes: *k*_*c*,*s*_ = *k*_*r*,*s*_ = *k*_*s*_. Then the minimal model of a single cell cycle can be represented by the following system of ordinary differential equations

7 $$\{\begin{array}{c}\frac{dV}{dt}=\frac{{k}_{s}}{{\alpha }_{r}}{t}_{e}{{\bf{S}}}_{r}V,\\ \frac{dc}{dt}={k}_{s}{t}_{e}({{\bf{S}}}_{c}-\frac{1}{{\alpha }_{r}}{{\bf{S}}}_{r}C),\end{array}C=(c+{t}_{e}({{\bf{S}}}_{c}+{{\bf{S}}}_{r})),{t}_{e}=\frac{1}{\sqrt{\frac{{k}_{s}}{{\alpha }_{r}}{{\bf{S}}}_{r}+{(\frac{{k}_{s}}{2})}^{2}}+\frac{{k}_{s}}{2}}.$$

Thus, the phenomenon of phenotypic multiplicity of a single cycle of the simplest self-reproducing system is reduced to the analysis of the number of positive solutions of equation

8 $$\frac{{{\bf{S}}}_{c}}{C}-\frac{{{\bf{S}}}_{r}}{{\alpha }_{r}}=0.$$

Evidently, the answer to this question depends on the type of functions **S**_*x*_, *x* = *c*, *r*. Functions **S**_*x*_ describe nonlinear processes of formation of active complexes between the generalized synthesis factor and its binding sites located on the corresponding mRNA molecules (analogues are SD sites). Taking into account the biochemical nature of these processes, as well as the basic principles of genetic regulation of transcription and translation, we can assume that **S**_*x*_ functions are sufficiently smooth, non-negative, bounded from above by functions with argument *c*. The functional purpose of the processes described by these functions allows to assume that **S**_*x*_ functions are unimodal (if there is a negative regulation) or monotonically increasing (if there is no negative regulation).

#### Types of functions **S**_*c*_, **S**_*r*_

To analyze possible forms of **S**_*x*_ function, let us consider the simplest one-stage formation of the initiator complex between the synthesis factor *s* and the initiation site *m*_*x*_

9 $${m}_{x}+s\rightleftarrows {m}_{x,c},x=c,r,$$

In equation (9), *m*_*x*_ is the concentration of free initiation sites (prototypes are promoters and mRNA SD sites); *s* denotes the concentration of free synthesis factors.

Let us carry out standard calculations, which are analogous to those carried out in the derivation of the famous formula describing the rate of the enzymatic reaction^47,48^. To do so, let us assume that all molecules of the synthesis factor (*c*) that are not ≪engaged≫ in the elongation are quickly redistributed between the pools of free molecules and pools of molecules of the *m*_*x*,*c*_ complexes. As a result, we obtain the dynamic conservation law

10 $$s+{m}_{c,c}+{m}_{r,c}=c.$$

Then, considering equation (9) in equilibrium and taking into account the conservation law equation (10), we obtain

11 $${{\bf{S}}}_{x}={k}_{x}{m}_{x,c},{m}_{x,c}=\frac{c}{{K}_{c}+c},x=c,r.$$

where, *k*_*x*_ is the parameter of the synthesis rate and *K*_*x*_ is the dissociation constant, *x* = *c*, *r*. Equation (9) circuit is the simplest because it does not consider ribosomal subunits, the multistage assembly of the functional ribosomal complex and many other known details, including the fact that parameters in equation (11) are not constant values, but depend on current concentrations of a number of intracellular substances, including the energy resources.

Substituting the equalities from equation (11) in equation (8) we obtain

12 $$\frac{{k}_{c}c}{{K}_{c}+c}\frac{1}{C}-\frac{1}{{\alpha }_{r}}\frac{{k}_{r}c}{{K}_{r}+c}=0,C=(c+\frac{\frac{{k}_{c}c}{{K}_{c}+c}+\frac{{k}_{r}c}{{K}_{r}+c}}{\sqrt{\frac{{k}_{s}}{{\alpha }_{r}}\frac{{k}_{r}c}{{K}_{r}+c}+{(\frac{{k}_{s}}{2})}^{2}}+\frac{{k}_{s}}{2}})$$

Direct calculations show the existence of sets of parameter values for which equation (12) has three positive roots (Fig. 1А).

Let us eliminate the elongation stage from the model (7). We obtain the system

13 $$\{\begin{array}{c}\frac{dV}{dt}=\frac{1}{{\alpha }_{r}}{{\bf{S}}}_{r},\\ \frac{dc}{dt}=c(\frac{{{\bf{S}}}_{c}}{c}-\frac{1}{{\alpha }_{r}}{{\bf{S}}}_{r}).\end{array}$$

As a result, we obtain the stationary equation

14 $$\frac{{{\bf{S}}}_{c}}{c}-\frac{{{\bf{S}}}_{r}}{{\alpha }_{r}}=0.$$

Substituting ***S***_*c*,*r*_ from equation (11) we obtain the equation

15 $$\frac{{k}_{c}}{{K}_{c}+c}-\frac{c}{{\alpha }_{r}}\frac{{k}_{r}}{{K}_{r}+c}=0.$$

After performing simple calculations, we find the only positive root

$$\,c=\frac{1}{2}(\sqrt{{\beta }^{2}+4\frac{\alpha {k}_{c}{K}_{r}}{{k}_{r}}}-\beta ),\beta ={K}_{c}-\frac{\alpha {k}_{c}}{{k}_{r}}.$$

Therefore, in the absence of the elongation stage, the one-stage initiation in factor synthesis described by equations (9–11) does not provide molecular prerequisites for phenotypic multiplicity of a single cell cycle.

It follows that elongation stage is absolutely necessary for the formation of phenotypic multiplicity in the case of simplest one-stage synthesis initiation described in equation (9). However, it is known that mechanisms of formation of synthesizing complexes inherent in living systems have a more complex molecular nature.

Let us derive functions **S**_*c*_ and **S**_*r*_ based on the two well-known facts: f1 – mechanisms of transcription/translation initiation are multistage (translation initiation consists of at least two consecutive stages); f2 – ribosomes and RNA polymerases are multisubunit protein complexes.

Let us write out the simplest variant that derives from f1. The simplest two-stage scheme of synthesis initiation, corresponding to f1, has the following form

16 $$\begin{array}{c}{m}_{x}+s\rightleftarrows {m}_{x,c},{m}_{x,c}+s\rightleftarrows {m}_{x,cc},x=c,r,\\ s+{m}_{c,c}+2{m}_{c,cc}+{m}_{r,c}+2{m}_{r,cc}=c.\end{array}$$

From it, using standard assumptions and calculations, we obtain the following expression

17 $${{\bf{S}}}_{x}=\frac{{k}_{x}{c}^{2}}{{K}_{x,1}{K}_{x,2}+{K}_{x,1}c+{c}^{2}},x=c,r.$$

Here, *k*_*x*_ and *K*_*x*,1_, *K*_*x*,2_, *x* = *c*, *r*, have standard meanings. From equation (14), with allowance for equation (17), we get the following cubic equation to search for the equilibrium concentration *c*

18 $$\begin{array}{c}{c}^{3}-{a}_{2}{c}^{2}+{a}_{1}c-{a}_{0}=0,\\ {a}_{0}=\frac{{\alpha }_{r}{k}_{c}}{{k}_{r}}{K}_{r,1}{K}_{r,2},{a}_{1}={K}_{c,1}{K}_{c,2}-\frac{{\alpha }_{r}{k}_{c}}{{k}_{r}}{K}_{r,1},{a}_{2}=\frac{{\alpha }_{r}{k}_{c}}{{k}_{r}}-{K}_{c,1}.\end{array}$$

Let us now write out the simplest variant that derives from f2.

We write out a mechanism in which synthesis functions **S**_*x*_ are formed as a result of the sequential formation of the dimeric functional molecule *s* and active synthesizing complexes with the involvement of *s*:

19 $$\begin{array}{c}a+a\rightleftarrows s,\,\,\,{m}_{x}+s\rightleftarrows {m}_{x,c},x=c,r,\\ a+2s+2{m}_{x,c}=c.\end{array}$$

Again, using standard transformations we obtain the following expression

20 $${{\bf{S}}}_{x}=\frac{{k}_{x}{c}^{2}}{{K}_{x}^{2}+{c}^{2}},x=c,r.$$

From equation (14), with allowance for equation (20), we get the following cubic equation to search for the equilibrium concentration *c*

21 $${c}^{3}-{a}_{2}{c}^{2}+{a}_{1}c-{a}_{0}=0,{a}_{0}=\frac{{\alpha }_{r}{k}_{c}}{{k}_{r}}{K}_{r}^{2},{a}_{1}={K}_{c}^{2},{a}_{2}=\frac{{\alpha }_{r}{k}_{c}}{{k}_{r}}.$$

From the form of equations (18 and 21) it follows that each of them has at least one positive solution. However, for certain parameter values, equations (18 and 21) can have up to three (considering a multiplicity) solutions. There are three positive solutions when the following inequalities are fulfilled

22 $$\begin{array}{l}{a}_{1} > 0,\,{a}_{2} > 0,\,{a}_{2}^{2}\ge 3{a}_{1},\\ {({a}_{2}+\sqrt{{a}_{2}^{2}-3{a}_{1}})}^{2}({a}_{2}-2\sqrt{{a}_{2}^{2}-3{a}_{1}})\\ \le 27{a}_{0}\\ \le {({a}_{2}-\sqrt{{a}_{2}^{2}-3{a}_{1}})}^{2}({a}_{2}+2\sqrt{{a}_{2}^{2}-3{a}_{1}}),\end{array}$$

Specific examples are given in Fig. 2B,C.

## Electronic supplementary material

Supplementary Information
