# Supplementary material for: Persister Cells – a Plausible Outcome of Neutral Coevolutionary Drift
Source: Sci Rep. 2018 Sep 25;8:14309. doi: 10.1038/s41598-018-32637-2 (PMC6156226; doi:10.1038/s41598-018-32637-2)
Supplement: Supplementary file 1 — Supplementary Information [file 41598_2018_32637_MOESM1_ESM.pdf]

PERSISTER CELLS – A PLAUSIBLE OUTCOME OF NEUTRAL COEVOLUTIONARY DRIFT

Tamara M. Khlebodarova, Vitaly A. Likhoshvai

Supplementary Information

SI1. The conditions that induce phenotypic multiplicity of the cell cycle. Let us describe the rate of change in protein concentration during the cell cycle.

Let us denote a certain protein and its concentration by *p*. Then the rate of change in the given protein concentration due to its synthesis is described by the general form equation

$$\frac{dp}{dt} = \mathbf{S}_p(B). \tag{S1}$$

The rate of change in protein *p* concentration in Eq. S1 we denote by $\mathbf{S}_p$, indicating in an explicit form that it depends on the concentration of proteins of the group *B* (self-reproducing proteins such as ribosomes, RNA polymerase, etc). Let us denote the cell volume by *V* and the cell volume growth – by **Y**. The totality of proteins whose concentration determines the growth rate of the cell volume we denote by *H*. Then the cell growth rate is described by the general form equation

$$\frac{dV}{dt} = \mathbf{Y}(H)V, \tag{S2}$$

and the dilution rate of protein *p* concentration due to cell volume growth is described with equality

$$\frac{dp}{dt} = -\frac{dV}{dt}\frac{1}{V}p = -\mathbf{Y}(H)p. \tag{S3}$$

As a result, the rate of change in protein concentration, taking into account its synthesis (Eq. S1) and dilution (Eq. S3), can be written in the form

$$\frac{dp}{dt} = \mathbf{S}_p(B) - \mathbf{Y}(H)p. \tag{S4}$$

We shall note that protein degradation is not considered in Eq. S4, which is an important element involved in the cell cycle regulation of modern cells, considering the external and internal environmental factors. But, for simplicity, we excluded protein degradation from consideration, although degradation reduces protein concentration and under certain conditions can lead to complex chaotic changes in intracellular protein concentration (Likhoshvai et al., 2016).

We assume that if we can identify the mechanism for the formation of phenotypic multiplicity of the cell cycle, which does not include degradation processes, this would mean that protein degradation is not the primary phenomenon-forming factor, although it can play a similar role under certain conditions.

From *H* we choose a protein, which is consumed for the cell growth and denote it by *r*. Assume there is at least one such protein. This assumption is quite realistic. For example, in modern *E. coli* cells it is Lpp membrane protein (Inouye et al., 1972); such proteins exist in *S. typhimurium*, *B. subtilis*, *Mycobacterium tuberculosis*, etc. In our opinion, this statement is valid for the majority of cells (if not all) and therefore does not limit the generality of reasoning in any way. The consumption rate for such protein during cell volume growth is described by the equation

$$\frac{dr}{dt} = -\alpha_r \mathbf{Y}(H). \tag{S5}$$

Where, α_r is the stoichiometric coefficient equal to the number of protein *r* molecules consumed for the cell volume growth during one cycle. Then the general equation describing the rate of change in protein *r* concentration is written in the form

$$\frac{dr}{dt} = \mathbf{S}_r(B) - \alpha_r \mathbf{Y}(H) - \mathbf{Y}(H) r \,. \qquad \text{(S6)}$$

Let us assume that cell growth is sufficiently effective in a sense that newly synthesized protein *r* molecules are rapidly consumed during cell growth, that is, the rate of cell growth is approximately equal to the rate of protein *r* synthesis

$$\mathbf{S}_r(B) \approx \alpha_r \mathbf{Y}(H). \qquad \text{(S7)}$$

Equality (S7) allows to exclude the law of cell volume growth from consideration and rewrite Eqs. S2 and S4 in the form

$$\frac{dV}{dt} = \frac{1}{\alpha_r} \mathbf{S}_r(B) V \,, \qquad \text{(S8)}$$

$$\frac{dp}{dt} = \mathbf{S}_p(B) - \frac{\mathbf{S}_r(B)}{\alpha_r} p \,. \qquad \text{(S9)}$$

Thus, we obtain a system of Eqs. S8,S9, which describes the rate of change in protein concentration during the cell cycle.

Let us consider the cell cycle as a dynamic process. A cell born at the time t_0 develops for some time *T*, after which it divides and two daughter cells appear instead of the original (mother) cell at the time $t_1=t_0+T$. Each daughter cell at the time of division receives all the necessary components for subsequent growth. We assume that division occurs in a simplest symmetrical way: daughter cells receive exactly half of all molecules at the time of division and the daughter cell volume is exactly half the volume of the mother cell just before division. Repeatedly, each cell undergoes growth and subsequent division. During the cell cycle, the rate of change in protein concentration varies according to Eqs. S8 and S9.

Now let us consider a cell cycle, in which daughter cells are identical to the mother cell at the time of its birth. That is, cell cycle of the daughter cell repeats the cell cycle of the mother cell. In other words, the cell cycle represents a stationary system. It can be expressed in the form of equalities

$$\begin{cases} \dfrac{dx(t+T)}{dt} = \dfrac{dx(t)}{dt}, \\ x(t+T) = x(t), x \in B. \end{cases} \qquad \text{(S10)}$$

Where, *t* is any time point of the cell cycle that is convenient to calculate according to an internal clock, taking the birth moment as 0.

It follows from Eqs. S10 that curves $x(t)$ are cyclic. Hence, for each *x* there is $0 \le t_x \le T$, for which

$$\frac{dx(t_x)}{dt} = 0. \qquad \text{(S11)}$$

Let us now consider a protein that does not belong to the group *B*. Let us denote its concentration by *m*. Then, from Eq. S11, for this protein we obtain the following equality at the point t_m

$$\frac{\mathbf{S}_r(B)}{\alpha_r} m = \mathbf{S}_m(B). \qquad \text{(S12)}$$

It follows from Eq. S12 that if functions $\mathbf{S}_r$ and $\mathbf{S}_m$ do not depend on *m*, then *m* is uniquely expressed in terms of concentration of proteins from the group *B*. That is, if protein *m* does not belong to the group *B*, its synthesis and dilution can act as factors that engender phenotypic multiplicity of the cell cycle only if there are specific feedback regulatory loops in the process of its synthesis (Shearwin, 2009;

Klumpp et al., 2009). Otherwise, synthesis and dilution of proteins not belonging to the group *B* are not capable of generating phenotypic multiplicity.

Let us now consider a protein from the group *B*. We denote its concentration by *c*. Then, at the point t_c we have the equality

$$\frac{\mathbf{S}_r\left(\overline{B},c\right)}{\alpha_r}c=\mathbf{S}_c\left(\overline{B},c\right),\overline{B}=B\setminus c\,. \tag{S13}$$

In Eq. S13, *B**c* denotes a group of proteins *B* excluding protein *c*. It can be seen that functions $\mathbf{S}_r$ and $\mathbf{S}_c$ automatically depend on *c* and the number of solutions to equation S13 depends significantly on the form of functions $\mathbf{S}_x\left(\overline{B},c\right),x=c,r$. It follows that synthesis and dilution of proteins from group *B* (RNA polymerases, ribosomal proteins) can potentially act as factors that engender phenotypic multiplicity of the cell cycle.

SI2. Analysis of the behavior of the adaptability functional W in the model (13) (see the main text of the article)**.** We assume that evolutionary adaptation is directed towards increasing the specific growth rate of the «cell», the metabolism of which is in equilibrium. Therefore, for the model (13), the adaptability functional has the following form

$$\mathbf{W}(c)=\frac{\mathbf{S}_r(c)}{\alpha_r}, \tag{S14}$$

where, *c* is a positive root of Eq. 14, which corresponds to a stable steady-state of Eqs. 13 (see the main text of the article). If Eq. 14 has more than one steady-state, then the root value, for which the value of **W** is higher, is taken as *c*. Let us study the behavior of **W** (Eq. S14) for the synthesis rate functions described by Eq. 20. In this case Eq. S14 is written as

$$\mathbf{W}(c)=\frac{\mathbf{S}_r(c)}{\alpha_r},\mathbf{S}_x=k_x\frac{c^2}{K_x^2+c^2},x=c,r. \tag{S15}$$

We see that **W** is directly dependent on the values $\frac{k_r}{\alpha_r}$, K_r and *c*. Since *c* is the solution of Eq. 21 (see the main text of the article), then **W** indirectly depends on k_c and K_c. Let us carry out the analysis for the fixed value α_r. Then we have four evolving parameters k_c,K_c,k_r,K_r.

First, we analyze the behavior of the adaptability functional **W** (Eq. S15) as a function of the parameter values k_c and K_c.

We have

$$\frac{\partial}{\partial c}\mathbf{W}=\frac{1}{\alpha_r}\frac{\partial\mathbf{S}_r}{\partial c}=2k_rc\frac{K_r^2}{\left(K_r^2+c^2\right)^2}>0\,.$$

Therefore, if Eq. 14 (see the main text of the article) has more than one positive root, then **W** takes a maximum value at the maximum root. Therefore, we are interested in the sign of the derivative $\frac{\partial\mathbf{W}}{\partial k},k=k_c,K_c$, at the maximum root.

We have

$$\frac{\partial}{\partial k}\mathbf{W}=\frac{1}{\alpha_r}\frac{\partial\mathbf{S}_r}{\partial c}\frac{\partial c}{\partial k},\mathbf{W}=\frac{\mathbf{S}_r}{\alpha_r}=\frac{\mathbf{S}_c}{c}.$$

A distinctive property of the maximum root is its unconditional existence for any fixed set of parameter values and the validity of inequality

$$\frac{\partial}{\partial c}\left(\frac{\mathbf{S}_r}{\alpha_r}\right) \geq \frac{\partial}{\partial c}\left(\frac{\mathbf{S}_c}{c}\right).$$

Let us note that $\frac{\partial}{\partial k_c}\left(\frac{\mathbf{S}_c}{c}\right) = \frac{c}{\left(K_r^2 + c^2\right)} > 0$. Hence, the maximum root of Eq. 14 (see the main text of the article) grows with increasing k_c. Equivalently, the maximum root of Eq. S13 increases with decreasing K_c. Consequently, we conclude that value of the adaptability functional **W** increases with decreasing K_c and increasing k_c

Let us now demonstrate that if $K_c \leq K_r$, then Eq. 14 has exactly one positive root and its value decreases with increasing k_r and decreasing K_r. We rewrite Eq. 14 in the equivalent form $\alpha_r \frac{k_c}{k_r} = F(c), F(c) = c\frac{K_c^2 + c^2}{K_r^2 + c^2}$.

We have

$$\frac{\partial}{\partial c}F(c) = \frac{K_c^2 + c^2}{K_r^2 + c^2} + 2c^2 \frac{K_r^2 - K_c^2}{\left(K_r^2 + c^2\right)^2} > 0.$$

Hence, $F(c)$ is an increasing function with respect to c. Since $F(0) = 0, F(\infty) = \infty$, then equation $\alpha_r \frac{k_c}{k_r} = F(c)$ has exactly one positive root.

$$\frac{d}{dc}\left(\frac{K_r^2 + c^2}{c\left(K_c^2 + c^2\right)}\right)\frac{dc}{dk_r} = \frac{1}{\alpha_r k_c} \Rightarrow sign\frac{dc}{dk_r} = sign\left(\frac{2c^2\left(K_c^2 - K_r^2\right) - \left(K_r^2 + c^2\right)\left(K_c^2 + c^2\right)}{c\left(K_c^2 + c^2\right)}\right) = -1 < 0.$$

Correspondingly, we have

$$\forall K_r \geq K_c : \mathbf{W}(c(,k_c, K_c, k_r, K_r), k_r, K_r) \leq \mathbf{W}(c(,k_c, K_c, k_r, K_c), k_r, K_c).$$

Note that biochemical nature of the parameters k_c, K_c, k_r, K_r implies that they have a minimum and maximum boundaries of physiologically acceptable values, within which they can change in the course of evolution. Moreover, the identical nature of the synthesis of factors allows us to assume that parameter pairs k_c, k_r and K_c, K_r have the same boundaries: $0 \leq k_{min} \leq k_c, k_r \leq k_{max} < \infty$ and $0 < K_{min} \leq K_c, K_r \leq K_{max} \leq \infty$. With no loss of generality, we can assume that k_{min} ~0 (value of the initiation rate constant can be arbitrarily small); k_{max} <∞ (value of the initiation rate constant can not be arbitrarily large due to the physical limitations of the rate of molecular processes); K_{max} ~∞ (interaction between the synthesis factor and its target sites can be arbitrarily weak); 0 < K_{min} (interaction between the synthesis factor and its target sites can not be arbitrarily effective due to the physical limitations of the rate of molecular processes).

It is also obvious that there are objective physical limitations to the unlimited growth of the parameter c value. The simplest justification of this statement lies in a physical fact that in any finite volume there can be a finite (may be large but finite) number of molecules having nonzero volume. In fact, for any type of molecule, the real physiological boundary is much smaller, since there are thousands of molecules in a cell and all of them must function properly. Excessive density of molecules in a limited volume physically limits their mobility and negatively affects the reaction rate. As a result, we can assume that in the course of evolution the concentration value of the resource factor can not exceed a certain limiting value $c \leq c_{fis}$.

Let us consider the case in which the maximum root of the system Eq. 21 (the main text of article) for any fixed values k_r and K_r does not exceed c_{fis}. Since with fixed values k_r and K_r function $\mathbf{W}(c(k_c, K_c, k_r, K_r), k_r, K_r)$ increases with increasing k_c and decreasing K_c, then maximum value of the function for fixed k_r and K_r is reached at the boundary of the physiological variability of these parameters: $k_c = k_{max}$ and $K_c = K_{min}$. But, since $c=c(k_{max}, K_{min}, k_r, K_r)$ decreases and the value of $\mathbf{W}(c, k_r, K_r)$ increases with increasing k_r and decreasing K_r, then maximum value of the function is $\mathbf{W}(c(k_{max}, K_{min}, k_{max}, K_{min}), k_{max}, K_{min})$ and $c\left(k_{max}, K_{min}, k_{max}, K_{min}\right) = \alpha_r$.

Suppose now that the maximum root of the system (S26) for some fixed values k_c, k_r and K_c, K_r is greater than c_{fis}: $c(k_c, K_c, k_r, K_r) > c_{fis}$. Then, for fixed values k_r and K_r the maximum value $\mathbf{W}(c(k_c, K_c, k_r, K_r), k_r, K_r)$ is attained at those k_c and K_c, for which the maximum root is equal to the physiological maximum: $c(k_c, K_c, k_r, K_r) = c_{fis}$. Note that if k_c, K_c belong to the physiological area, then the pair of values $(k_c \frac{K_{min}^2 + c^2}{K_c^2 + c^2}, K_{min})$ also lies in the physiological area. Because $\left(k_c \frac{K_{min}^2 + c^2}{K_c^2 + c^2}\right) \cdot \frac{c^2}{K_{min}^2 + c^2} = k_c \frac{c^2}{K_c^2 + c^2}$, then $c(k_c, K_c, k_r, K_r) = c(k_c \frac{K_{min}^2 + c^2}{K_c^2 + c^2}, K_{min}, k_r, K_r) = c_{fis}$ and $\mathbf{W}(c(k_c, K_c, k_r, K_r), k_r, K_r) = \mathbf{W}(c(k_c \frac{K_{min}^2 + c^2}{K_c^2 + c^2}, K_{min}, k_r, K_r), k_r, K_r)$. Because $K_r \geq K_{min}$, then as value k_r increases and value K_r decreases we get a decrease of $c(k_c \frac{K_{min}^2 + c^2}{K_c^2 + c^2}, K_{min}, k_r, K_r)$ and an increase of $\mathbf{W}(c(k_c \frac{K_{min}^2 + c^2}{K_c^2 + c^2}, K_{min}, k_r, K_r), k_r, K_r)$. Therefore, the maximum of the adaptability functional is achieved when $\mathbf{W}(c(\bar{k}_c, K_{min}, k_{max}, K_{min}), k_{max}, K_{min}), \bar{k}_c = k_c \frac{K_{min}^2 + c^2}{K_c^2 + c^2}$, $c(\bar{k}_c, K_{min}, k_{max}, K_{min}) < c_{fis}$.

Because $\bar{k}_c < k_{max}$, then for any $\bar{k}_c \leq k_c \leq k_{max} \Rightarrow$
$\mathbf{W}(c(k_c, K_{min}, k_{max}, K_{min}), k_{max}, K_{min}) > \mathbf{W}(c(\bar{k}_c, K_{min}, k_{max}, K_{min}), k_{max}, K_{min})$.

Therefore, maximum of the adaptability functional $\mathbf{W}$ is achieved when $k_c = k_{max}$, $c(k_{max}, K_{min}, k_{max}, K_{min}) = \alpha_r$ or $c(k_c, K_{min}, k_{max}, K_{min}) = c_{fis}$, $k_c = k_r \frac{c_{fis}}{\alpha_r}$. It is obvious that the second possibility can be realized only for $\frac{c_{fis}}{\alpha_r} \leq 1$. Therefore, if $\frac{c_{fis}}{\alpha_r} \geq 1$, there is a single point at which $\mathbf{W}$ has the maximum value: $\mathbf{W}_{max} = \mathbf{W}(\alpha_r, k_{max}, K_{min}, k_{max}, K_{min})$.

If $\frac{c_{fis}}{\alpha_r} < 1$, then $\mathbf{W}_{max} = \mathbf{W}(c_{fis}, \frac{c_{fis}}{\alpha_r} k_{max}, K_{min}, k_{max}, K_{min})$. Let us take $\frac{c_{fis}}{\alpha_r} k_{max} \leq k_c \leq k_{max}$ and assume that $K_c = \sqrt{\frac{\alpha_r k_c}{c_{fis} k_{max}}\left(K_{min}^2 + c_{fis}^2\right) - c_{fis}^2} \geq K_{min}$. Then $\mathbf{W}_{max} = \mathbf{W}(c_{fis}, k_c, K_c, k_{max}, K_{min})$. That is, values of the parameters k_c, K_c can be any in the interval $\frac{c_{fis}}{\alpha_r} k_{max} \leq k_c \leq k_{max}$.

It follows from the above analysis that the adaptability functional **W** has a single global maximum. Regardless of the value of concentration *c*, the maximum is realized in the range of parameter values, which includes $K_c = K_r = K_{min}$. Values of the parameters k_c, k_r are determined by the ratio α_r/c_{fis} and physiological boundaries $k_c, k_r \le k_{max}$. Under certain conditions, the maximum value of the adaptability functional **W** is realized not at a single point, but in some nontrivial parametric region, which should be called the region of neutrality. In the areas of neutrality, the parameter values can be chosen arbitrarily, considering the fulfillment of certain relations.

Let us interpret the obtained results given the paradigm that the evolutionary selection of the most adopted cells leads to an increase in the value of the adaptability functional **W**. Let us consider two cases:

1) If $\frac{\alpha_r}{c_{fis}} \le 1$, then evolution leads to an increase/decrease in the values of the parameters $k_c, k_r / K_c, K_r$ until they reach the maximum/minimum values: $k_c, k_r \to k_{max} / K_c, K_r \to K_{min}$.

Additionally, Eq. 22 (the main text of article) has only one positive root at the limit point, which means that region of phenotypic multiplicity does not lie in the attractive area from an evolutionary point of view.

2) If $\frac{\alpha_r}{c_{fis}} > 1$, then the parametric region of the maximal adaptability is not a single point, but represents some non-trivial parametric manifold.

Let us consider cells that are in a state of maximal adaptability. These cells continue to grow, divide, and accumulate mutations. Most frequent mutations (single or multiple) are mutations that lower the adaptability of the cell. Therefore, they are eliminated during selection. But from time to time cells bearing compensated mutations would appear. Since $\frac{\alpha_r}{c_{fis}} > 1$, such mutations include the ones that change values of parameters k_c and K_c for $k_{c,mut}$ and $K_{c,mut}$ in a consistent way in a sense of fulfilling the equality

$$\frac{k_c}{K_c^2 + c^2} = \frac{k_{c,mut}}{K_{c,mut}^2 + c^2}. \tag{S16}$$

Then all cells with parameters $k_{x,mut}$ and $K_{x,mut}$ possess the same maximal adaptability as the cells with parameters k_x and $K_x, x = c, r$. That is, cells carrying such combinations of mutations are not subjected to negative selection and all cells carrying these mutations have absolutely identical chances to survive or be eliminated from the population. Therefore, such coupled mutations are neutral. Accordingly, in a developing cell population, cells with any set of parameter values from the physiological range of parameters of the non-changing (maximal) adaptability functional **W** will appear over time.

Therefore, for $\frac{\alpha_r}{c_{fis}} > 1$, there exists a nondegenerate parametric area within which a coevolutionary neutral drift of coupled mutations occurs.

In this connection, the question arises whether regions of coevolutionary neutral drift of coupled mutations can contain subregions in which multiple phenotypes of a single cell cycle can be realized.

Let us study the properties of the parametric region of the coordinated neutral drift for

$$\frac{\alpha_r}{c_{fis}} > 1. \tag{S17}$$

Then, the maximum value of **W** is reached at c_{fis} and $k_r = k_{max}, K_r = K_{min}$:

$$\mathbf{W}_{max} = \frac{c_{fis}^2}{\alpha_r}\frac{k_{max}}{K_{min}^2 + c_{fis}^2}. \quad \text{(S18)}$$

A parametric region of the coordinated neutral drift represents an interval [$k_{c,max}$, k_{max}], $k_{c,max}=c_{fis}k_{max}/\alpha_r$., Value of the parameter k_c can be chosen arbitrarily from this interval. Assume that $K_c = \sqrt{\frac{k_c}{k_{c,max}}\left(K_{min}^2 + c_{fis}^2\right) - c_{fis}^2}$ and lies in the interval [K_{min}, $K_{c,max}$], $K_{c,max} = \sqrt{\frac{\alpha_r}{c_{fis}}\left(K_{min}^2 + c_{fis}^2\right) - c_{fis}^2}$.

Let us investigate the conditions of existence of three roots for the following equation

$$\begin{gathered} c\left(K_{c,max}^2 + c^2\right) - \alpha_r\left(K_{min}^2 + c^2\right) = 0, \\ c^3 - a_2c^2 + a_1c - a_0 = 0, a_0 = \alpha_r K_{min}^2, a_1 = K_{c,max}^2, a_2 = \alpha_r. \end{gathered} \quad \text{(S19)}$$

We take into account that c_{fis} is the root of Eq. 21 from the main text. We have

$$c^3 - a_2c^2 + a_1c - a_0 = (c^2 - b_1c + b_0)(c - c_{fis}),$$

$$b_1 = \alpha_r - c_{fis}, b_0 = \frac{\alpha_r}{c_{fis}}K_{min}^2,\ c_{1,2} = \alpha_r - c_{fis} \pm \sqrt{\left(\alpha_r - c_{fis}\right)^2 - \frac{\alpha_r}{c_{fis}}K_{min}^2},$$

$$\left(\alpha_r - c_{fis}\right)^2 - \frac{\alpha_r}{c_{fis}}K_{min}^2 \ge 0 \Rightarrow \left(\frac{\alpha_r}{c_{fis}}\right)^2 - 2\left(1 + \frac{1}{2}\left(\frac{K_{min}}{c_{fis}}\right)^2\right)\left(\frac{\alpha_r}{c_{fis}}\right) + 1 \ge 0 \Rightarrow,$$

$$\left(\frac{\alpha_r}{c_{fis}}\right)_{1,2} = \left(1 + \frac{1}{2}\left(\frac{K_{min}}{c_{fis}}\right)^2\right) \pm \sqrt{\left(1 + \frac{1}{2}\left(\frac{K_{min}}{c_{fis}}\right)^2\right) - 1}.$$

Hence, the necessary condition of existence of three roots is the fulfillment of inequality

$$\left(\frac{\alpha_r}{c_{fis}}\right) \ge \left(\left(\frac{K_{min}}{2c_{fis}}\right) + \sqrt{1 + \frac{1}{4}\left(\frac{K_{min}}{c_{fis}}\right)^2}\right)^2 \quad \text{(S20)}$$

Thus, a non-trivial intersection between the neutrality line and the region of existence of three positive roots exists only if conditions S17 and S20 are satisfied.

A reasonable question arises whether these conditions are physiologically realistic. In our calculations we used experimental data obtained for the actively growing *E. coli* cell (Schaechter et al., 1962; Inouye and Shaw, 1972; Bremer and Dennis, 1996; Cowles et al., 2011; Dai et al., 2016). Value of the parameter α_r was estimated from the relative amount of Lpp that is consumed in the process of the *E. coli* cell wall construction during one cell cycle. Based on the data from (Inouye and Shaw, 1972), it was determined to be $\alpha_r \sim 5\cdot10^5$. The parameter c_{fis} was estimated from the number of free ribosomes competent of initiating translation and was determined to be $c_{fis} \sim 2\cdot10^4$-$1.5\cdot10^5$ unit/cell. We assumed that total number of ribosomes in the actively growing cell (cell cycle duration ~20 min) is ~$6\cdot10^4$ pieces/μm^3, cell volume is ~4-8 μm^3, and the number of ribosomes participating in the translation elongation comprises ~70-90% of the total number of ribosomes. Hence, $\alpha_r > c_{fis}$.

The condition for the inequality S34 depends on the value of the parameter K_{min}, which determines the effectiveness of the interaction between ribosomes and SD sites, and strongly depends on the structure of SD sites and surrounding regions. It can vary within very wide limits. It would not be a mistake to assume that for the most effectively translated mRNA molecules the dissociation constant *K*

is below the point at which 50% of the translation initiation is achieved. Therefore, we assume that K_{min}=10000 unit/cell. Whence, α_r~$5\cdot10^5$, c_{fis}~$2\cdot10^4$-$15\cdot10^4$, K_{min}=10^4 and we have

$$\alpha_r \sim 500000 \geq 32807.8 \div 160339 \sim c_{fis}\left(\left(\frac{K_{min}}{2c_{fis}}\right)+\sqrt{1+\frac{1}{4}\left(\frac{K_{min}}{c_{fis}}\right)^2}\right)^2,$$

that is, the inequality S20 is fulfilled.

Thus, parameters of the modern *E.coli* cell completely satisfy the conditions for the realization of phenotypic multiplicity.

SI3. The role of nutritional resource in the cell cycle phenotypic multiplicity formation. In this section we demonstrate that nutritional resource, being a source of cellular self-reproduction, is a natural factor that is capable of forming the conditions necessary for the realization of the phenotypic multiplicity of the cell cycle. To show this, let us include the nutritional resource into the model (Eqs 13) (the main text of the article) in a simplest linear form. Namely, we assume that the nutritional resource enters the cell and is consumed during the synthesis of factors. As a result, we get the following model

$$\begin{cases} \frac{d}{dt}V = z\frac{\mathbf{S}_r}{\alpha_r}V, \\ \frac{d}{dt}c = z\left(\frac{\mathbf{S}_c}{c}-\frac{\mathbf{S}_r}{\alpha_r}\right)c, \end{cases} \tag{S21}$$

$$z = \frac{k_{zin}}{\left(k_{zout}+\Delta_z\left(\mathbf{S}_c+\mathbf{S}_r\right)\right)}.$$

Where, z – current concentration of the resource, k_{zin} – rate constant for the resource flow into the area of consumption, k_{zout} – rate constant for the resource outflow from the area of consumption, Δ_z – unit cost of the factor synthesis process. For simplicity, we assume that processes of inflow and outflow/consumption of the resource are balanced.

In this case, the adaptability functional takes the form

$$\mathbf{W} = z\frac{\mathbf{S}_r}{\alpha_r}, \tag{S22}$$

and its value should be calculated for the positive stable root of equation

$$\frac{1}{\alpha_r}\mathbf{S}_r = \frac{1}{c}\mathbf{S}_c. \tag{S23}$$

A distinctive behaviour of the adaptability functional $\mathbf{W} = z\frac{\mathbf{S}_r}{\alpha_r}$ for the monotonically increasing and bounded from above function $\mathbf{S}_r$ and limited nutritional resource is the appearance of a global maximum for a finite c value, which follows directly from the representation

$$\mathbf{W} = \frac{k_{zin}}{\alpha_r}\frac{\mathbf{S}_r}{\left(k_{zout}+\Delta_z\left(\frac{c}{\alpha_r}+1\right)\mathbf{S}_r\right)}. \tag{S24}$$

If we additionally assume that $\mathbf{S}_r, \mathbf{S}_c$ are smooth functions of the positive argument c and $\frac{d}{dc}\left(\frac{1}{\mathbf{S}_r}\right)$ monotonically increases from -∞ to zero with c increasing from 0 to +∞, then it follows from equality

$$\frac{d}{dc}\mathbf{W} = -\frac{k_{zin}}{k_{zout}\alpha_r}\frac{\frac{d}{dc}\left(\frac{1}{\mathbf{S}_r}\right)+\frac{\Delta_z}{k_{zout}\alpha_r}}{\left(\frac{1}{\mathbf{S}_r}+\frac{\Delta_z}{k_{zout}\alpha_r}(c+\alpha_r)\right)^2} = 0,$$

that functional **W** (Eq. S24) has a single maximum and there is a decrease in c_{fis} value with increasing Δ_z, in which the given maximum is reached.

It is possible to calculate the position of c_{fis} maximum in an explicit form for a particular type of $\mathbf{S}_r$ (Eq. 20 from the main text). We have

$$\mathbf{S}_r = k_r\frac{c^2}{K_r^2+c^2} \Rightarrow \frac{1}{\mathbf{S}_r} = \frac{1}{k_r}+\frac{K_r^2}{k_r c^2} \Rightarrow \frac{d}{dc}\left(\frac{1}{\mathbf{S}_r}\right) = -\frac{2K_r^2}{k_r c^3}, \Rightarrow c^3 = \frac{2K_r^2 k_{zout}\alpha_r}{\Delta_z k_r}.$$

or

$$\frac{c_{fis}}{K_{min}} = \sqrt[3]{2\frac{k_{zout}\alpha_r}{k_{max}K_{min}\Delta_z}} \quad \text{(S25)}$$

Let us now study the number of positive solutions for Eq. 21 from the main text depending on the $\frac{\Delta_z}{k_{zout}}$ value. We shall carry out a study for a specific form of the functional **W** (Eq.S15). In this case, Eq. S25 represents a one-to-one correlation between c_{fis} and $\frac{\Delta_z}{k_{zout}}$. We rewrite Eq. S20 in a more convenient form

$$\left(\frac{\alpha_r}{K_{min}}\right)\left(\frac{K_{min}}{c_{fis}}\right) \geq \left(\frac{1}{2}\left(\frac{K_{min}}{c_{fis}}\right)+\sqrt{1+\frac{1}{4}\left(\frac{K_{min}}{c_{fis}}\right)^2}\right)^2 \Rightarrow$$

$$\sqrt{\left(\frac{\alpha_r}{K_{min}}\right)} \geq \sqrt{\left(\frac{K_{min}}{c_{fis}}\right)}\left(\frac{1}{2}+\sqrt{\left(\frac{c_{fis}}{K_{min}}\right)^2+\frac{1}{4}}\right) \Rightarrow$$

$$\alpha \geq F(\chi) = \frac{1}{\chi}\left(\frac{1}{2}+\sqrt{\chi^4+\frac{1}{4}}\right),\ \alpha = \sqrt{\left(\frac{\alpha_r}{K_{min}}\right)},\ \chi = \sqrt{\left(\frac{c_{fis}}{K_{min}}\right)}.$$

Let us find the minimum of the function $F(\chi)$. We have

$$\frac{d}{d\chi}F(\chi) = -\frac{1}{\chi^2}\left(\frac{1}{2}+\sqrt{\chi^4+\frac{1}{4}}\right)+\frac{1}{\chi}\frac{2\chi^3}{\sqrt{\chi^4+\frac{1}{4}}} = \frac{-\left(\frac{1}{2}\sqrt{\chi^4+\frac{1}{4}}+\chi^4+\frac{1}{4}\right)+2\chi^4}{\chi^2\sqrt{\chi^4+\frac{1}{4}}} \Rightarrow$$

$$-\left(\frac{1}{2}\sqrt{\chi^4+\frac{1}{4}}+\chi^4+\frac{1}{4}\right)+2\chi^4 = 0 \Rightarrow \chi^4-\frac{1}{4} = \frac{1}{2}\sqrt{\chi^4+\frac{1}{4}} \Rightarrow$$

$$x^2-\frac{1}{2}x+\frac{1}{16} = \frac{1}{4}x+\frac{1}{16}, x = \chi^4 \Rightarrow x = \frac{3}{4} \Rightarrow \chi = \sqrt[4]{\frac{3}{4}}.$$

Whence, $F_{\min} = F(\sqrt[4]{\frac{3}{4}}) = \frac{3}{2}\sqrt[4]{\frac{4}{3}}$.

Therefore, if $\frac{\alpha_r}{K_{min}} \le \frac{3}{2}\sqrt{3}$, then for any resource availability, there is only one root (Fig.S1, line 2 does not intersect the graph of the function *F*). If $\frac{\alpha_r}{K_{min}} > \frac{3}{2}\sqrt{3}$, then the number of positive solutions depends on the availability of the resource (Fig.S1, line 1 intersects the graph of the function *F*).

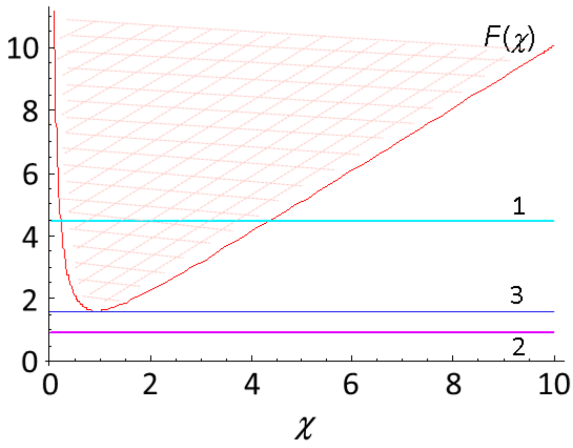

Figure S1. Graph of the function *F*(χ). Line 1 – α=4.47214 (plurality of steady-states is present in the interval $0.22 < \chi < 4.36 \xrightarrow{\chi = \sqrt[6]{2\frac{k_{zout}\alpha_r}{k_{max}K_{min}\Delta_z}}} 1.18\cdot 10^{-7} < \frac{\Delta_z}{k_{zout}} < 6.3$); line 2 – α=0.894427 (a single steady-state); line 3 touches the minimum $F_{\min} = \frac{3}{2}\sqrt[4]{\frac{4}{3}}$. Abscissa – χ value, ordinate – values of the function *F* and α in relative units. The shaded part of the graph corresponds to the bistability region.

In connection with the obtained result, we shall note that the question of the physiological expediency of the relation $\frac{\alpha_r}{K_{min}}$ has been discussed above. Let us recall that α_r has the meaning of the number of growth factor molecules consumed in the process of cell growth during single cell cycle, and K_{min} has the meaning of the concentration of the synthesis factor, at which ½ the maximum synthesis rate is reached. Above we have indicated that analogs of these parameters in the modern cell, most likely, have the following values: $\alpha_r \ge 5\times10^5$, $K_{min} < 10^5$, т.е., $\alpha_r > \frac{3}{2}\sqrt{3}K_{min}$. That is, conditions for realization of bistability are quite realistic from a biological point of view. Therefore, we further assume that $\alpha_r > \frac{3}{2}\sqrt{3}K_{min}$. In this case, in a certain range of resource availability, a plurality of phenotypes is observed. Let us find the boundaries of this range. We have

$$\alpha = \frac{1}{\chi}\left(\frac{1}{2} + \sqrt{\chi^4 + \frac{1}{4}}\right),\ \alpha = \sqrt{\left(\frac{\alpha_r}{K_{min}}\right)},\ \chi = \sqrt{\left(\frac{c_{fis}}{K_{min}}\right)},$$

$$\left(\alpha\chi - \frac{1}{2}\right)^2 = \chi^4 + \frac{1}{4} \Rightarrow \alpha^2\chi^2 - \alpha\chi + \frac{1}{4} = \chi^4 + \frac{1}{4} \Rightarrow$$

$$\chi^3 - \alpha^2\chi + \alpha = 0 \Rightarrow x^3 - x + a = 0, x = \sqrt{\left(\frac{c_{fis}}{\alpha_r}\right)}, a = \frac{K_{min}}{\alpha_r} \ll \frac{2}{\sqrt{27}}.$$

$$\Delta_{z,min} = \frac{2\alpha_r}{K_{min}k_{max}x_{max}^6} \text{ and } \Delta_{z,max} = \frac{2\alpha_r}{K_{min}k_{max}x_{min}^6}.$$

It corresponds to the location of the blue horizontal line (Fig. S1.) α=4.47 relative to the graph of the function $F(x)$, that is, the multiplicity is present in the interval $1.17 \cdot 10^{-7} \le \Delta_z \le 6.3$. A single solution is observed with a high degree of availability (area of excess: $\frac{\Delta_z}{k_{zout}} < 1.17 \cdot 10^{-7}$) and with a high degree of insufficiency of the resource (area of high deficit: $6.3 < \frac{\Delta_z}{k_{zout}}$).

Thus, we have clearly shown that a relative insufficiency of the nutritional resource is a condition for the realization of bistability, namely the phenotypic multiplicity of the cell cycle.

References

1. Bremer, H. & Dennis, P.P. Modulation of chemical composition and other parameters of the cell by growth rate. In: *Escherichia coli* and *Salmonella*: Cellular and Molecular Biology (eds. Neidhardt, F.C. et al.), **2**, 1553–1569 (Washington: ASM Press, 1996).
2. Cowles, C.E., Li, Y., Semmelhack, M.F., Cristea, I.M. & Silhavy TJ. The free and bound forms of Lpp occupy distinct subcellular locations in *Escherichia coli*. *Mol. Microbiol.* **79**, 1168-1181 (2011)
3. Dai, X. et al. Reduction of translating ribosomes enables *Escherichia coli* to maintain elongation rates during slow growth. *Nat. Microbiol.* **2**,16231 (2016).
4. Inouye, M., Shaw, J. & Shen, C. The assembly of a structural lipoprotein in the envelope of *Escherichia coli*. *J. Biol. Chem.* **247**, 8154-8159 (1972)
5. Klumpp, S., Zhang, Z. & Hwa, T. Growth rate-dependent global effects on gene expression in bacteria. *Cell* **139**, 1366-1375 (2009).
6. Schaechter, M., Williamson, J.P., Hood, J.R. Jr. & Koch, A.L. Growth, cell and nuclear divisions in some bacteria. *J. Gen. Microbiol* **29,** 421-434 (1962).
7. Shearwin, K. Slow growth leads to a switch. *Nat. Chem. Biol.* **5**, 784-785 (2009).
